# Abdominal muscle activation: An EMG study of the Sahrmann five-level core stability test

**DOI:** 10.1142/S1013702520500080

**Published:** 2020-03-10

**Authors:** Ebby Waqqash Mohamad Chan, Mohamad Shariff A. Hamid, Ali Md. Nadzalan, Eliza Hafiz

**Affiliations:** 1 Centre for Sport & Exercise Sciences, University of Malaya, 50603 Kuala Lumpur, Malaysia; 2 Faculty of Medicine, University of Malaya, 50603 Kuala Lumpur, Malaysia; 3 Faculty of Sports Science and Coaching, Universiti Pendidikan Sultan Idris, 35900 Tanjong Malim, Perak Darul Ridzuan, Malaysia; ebbywaqqash@hotmail.com; eliza@um.edu.my

**Keywords:** Abdominal muscles, muscle activity, surface EMG, core stability

## Abstract

**Background:**

Sahrmann five-level core stability test protocol has been used to evaluate the ability of the core muscles to stabilize the spine. However, validation studies on the Sahrmann protocol are limited.

**Objective:**

The purpose of this study was to compare the different levels of Sahrmann five-level core stability (levels 1–5) on the muscle activity of rectus abdominis (RA), external oblique (EO), and transverse abdominis/internal oblique (TrA/IO).

**Methods:**

Twenty-two asymptomatic male participants aged 21.36±1.59 years were recruited. Participants were instructed to perform maximum voluntary contraction (MVC) and five levels of Sahrmann five-level core stability test guided with a pressure biofeedback unit (PBU). The surface electromyography (EMG) data of each muscle during five levels of Sahrmann five-level core stability test were normalized as a percentage of MVC.

**Results:**

Results showed significant differences in the normalized EMGs of RA [$\chi^{2}$(4) = 64.80, p<0.001], EO [$\chi^{2}$(4) = 58.11, p<0.001], and TrA/IO [$\chi^{2}$(4) = 56.00, p<0.001] between the five levels of Sahrmann five-level core stability test. Post-hoc analysis revealed Sahrmann levels 5 and 3 have significantly higher abdominal EMG signals than levels 4, 2, and 1 (p<0.001).

**Conclusion:**

In conclusion, the Sahrmann five-level core stability test differs according to the level of Sahrmann tests. Significantly higher abdominal muscle activities were observed during levels 3 and 5. Therefore, the classification exchange in levels 3 and 4 of the Sahrmann five-level core stability test should be reconsidered in the future.

## Introduction

Core stability is defined as the “ability of the lumbopelvic-hip complex to return to equilibrium following a perturbation without buckling of the vertebral column”.^1^ There are 29 muscles attached to the lumbopelvic-hip complex.^2^ The muscles which were frequently mentioned in previous researches are the multifidus, which stabilizes the vertebral joints on each segmental level, and the transverse abdominis (TrA), which stabilizes the spine through the increment of intra-abdominal pressure.^3^ Other superficial trunk muscles that contribute to spinal stability include abdominal muscles [rectus abdominis (RA), abdominal internal oblique (IO), and abdominal external oblique (EO)] and paraspinal muscles (erector spinae and quadratus lumborum). According to Barr *et al.*,^3^ these superficial muscles are activated to provide additional stability during direction and load-specific activity to prevent unwanted spinal displacement. It is highlighted that the entire core muscles have to be co-activated from all angles and directions to enable all layers of core muscles, deep and superficial to be physically bound together enhancing spinal stability and stiffness to a higher degree.^4^

Lower extremity movement protocol with pressure biofeedback transducer has been widely used by researchers to evaluate core stability performance.^5,6,7^ One of the commonly used lower extremity movement protocols to measure core stability is the Sahrmann five-level core stability test protocol.^7^ Sahrmann^8^ initially suggested a lower abdominal muscle progression grading which consists of nine different lower extremity movement protocol maneuvers (nine-level ordinal scale, scored on a scale of 0.10–5.00). However, previous researchers have utilized a modified version which only adapted five maneuvers from the original Sahrmann protocol.^9,10,11,12^ The Sahrmann five-level core stability test consists of five different leg lowering maneuvers (levels 1–5) that progressively increase in difficulty.^7^ Details of the Sahrmann five-level core stability test was explained in Sec. 2.

Aggarwal *et al.*^9^ have examined the relationship of commonly used core stability tests; Sorensen test, prone plank test, side plank test, abdominal fatigue test, and Sahrmann five-level core stability test. Aggarwal *et al.*^9^ discovered that the Sahrmann’s test performance was only significantly correlated with the performance of prone plank test (*Rho*= 0.408; p=0.009). It was highlighted that the significant correlation of Sahrmann five-level core stability test with the prone plank test indicates that both tests specifically evaluated the core stability performance in the sagittal plane.^9^

Although the Sahrmann protocol may indirectly evaluate the ability of the core muscles to stabilize the spine, studies on validation of the Sahrmann protocol are limited.^7^ Therefore, there is a need to validate the Sahrmann five-level core stability test by comparing the muscle recruitment pattern amongst the different levels/maneuvers of Sahrmann five-level core stability test particularly the maximum voluntary contraction percentage (MVC %) of RA, EO, and TrA/IO.

## Materials and Methods

### Participants

Twenty-two asymptomatic male students from the Sports Centre took part in this study. The mean ± standard deviation of the age was 21.36±1.59 years, of their weight was 65.83±8.37 kg, of their height was 1.71±0.06 cm, of their waist circumference was 74.39±5.23 cm, of their hip circumference was 92.80±4.59 cm, of their body mass index was 22.59±2.2 kg/m^2^, and of their waist–hip ratio was 0.80±0.03. The participants were screened using the self-reported Nordic musculoskeletal questionnaire (NMQ). Individuals were excluded if they are having acute low back pain (LBP) (<7 days of LBP) or chronic low back pain (>12 months of LBP). Individuals with a waist circumference greater than 94 cm were also excluded to reduce surface electromyography (EMG) artifact due to adipose tissue.^13^ The University Malaya Research Ethics Committee (UMREC) granted ethical approval for this study (Reference No. UM. TNC2/UMREC-338) and all participants provided written informed consent before their participation.

### Data acquisition

**Fig. 1.**
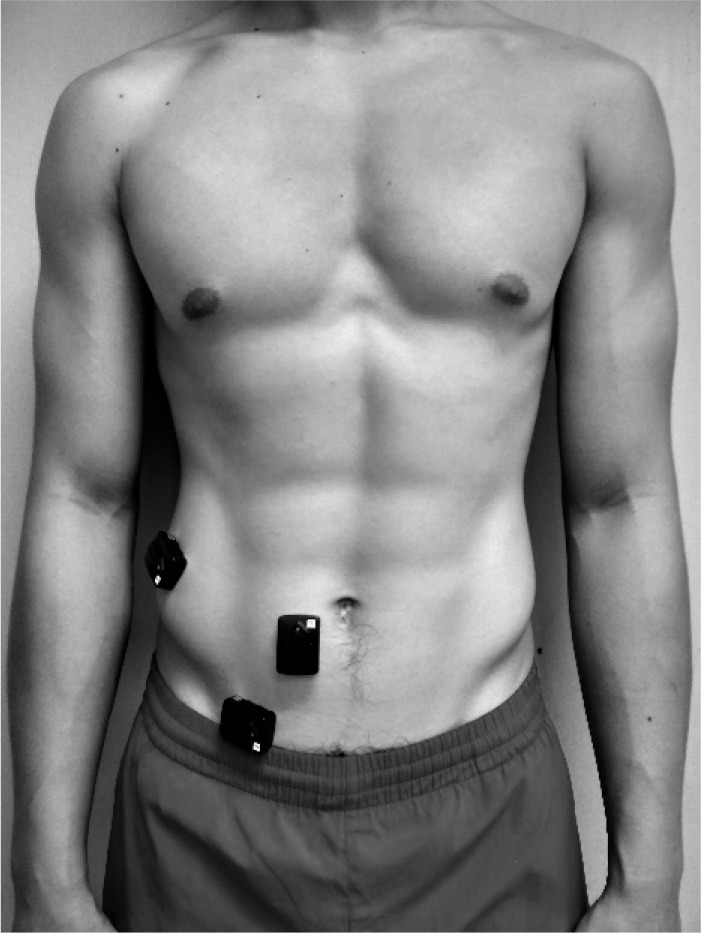
Experimental setup for surface EMG: RA, EO, and TrA/IO.

The wireless Trigno^TM^ system (Trigno, Delsys Inc., USA) was used to record and process the EMG signals of RA, EO, and TrA/IO. Surface electrodes were attached to the muscle fibers on the right-hand sides of the body based on the guidelines from the previous studies^14,15,16,17,18^ (see Fig. 1): (1) RA: 3 cm lateral to the umbilicus, (2) EO: 15 cm lateral to the umbilicus, and (3) TrA/IO: midway between the anterior iliac spine and symphysis pubis, above the inguinal ligament.

The raw EMG signals from the three muscle sites were amplified (common-mode rejection ratio (CMMR) of 130 dB, input impedance of 20,000 kΩ) using a differential amplifier (Trigno Wireless System, Delsys Inc., Boston, USA) and filtered using a Butterworth band-pass filter of bandwidth between 20 Hz and 450 Hz. The signals were converted from analog to digital at a sampling rate of 2,000 Hz for data processing and analysis (EMGworks 3.0, Delsys Analysis Software, Boston, USA). In order to minimize specific interfering frequency (i.e., electrical and radio frequencies), the EMG data acquisition was conducted in a controlled and contained laboratory.

### Normalization exercises

To standardize the action potential of each of the three abdominal muscles, all participants performed three MVCs against manual resistance as suggested by Vera-Garcia *et al.*^19^ The following are the MVC maneuvers for respective abdominal muscles:

(1) Rectus abdominis: Participants were in a sit-up posture positioned on a bench with the knees bent. They then attempted to flex the upper trunk in the sagittal plane while their thorax was manually braced by the experimenter.
(2) External oblique: Participants attempted to side bend the upper trunk in the frontal plane while they were in a side-lying position, with the knees bent, and thorax and arms were manually braced by the experimenter.
(3) Transverse abdominis/internal oblique: Participants maintained a right-side bridge position while a maximally resisted downward pressure on the pelvis was applied by the experimenter.

During the MVC testing, the participants were instructed to avoid any jerky contractions to decrease the chance of injury. To prevent muscle fatigue while the MVC was measured, each maneuver was performed in a random order, with each being held for 5 s and repeated twice with a 2-min rest between trials to prevent muscle fatigue.^13^

### Sahrmann five-level core stability test

All participants were trained to perform the five levels of the standard protocol of the Sahrmann five-level core stability test as suggested by Aggarwal *et al.*^9^ correctly before the measurement as described below. Each participant completed three trials at each of the five levels, with at least a 1-min rest between trials.

The inflatable pad of a stabilizer pressure biofeedback unit (PBU) (Chattanooga Group, Inc., Hixson, TN) was placed under the lumbar spine at approximately L4–L5. While the participant was lying in a crook-supine position, the PBU was inflated to 40 mmHg. Participants were told to perform the Sahrmann five-level test while drawing-in their abdomen to avoid pressure deviation of more than 10 mmHg. A deviation of pressure more than 10 mmHg indicates that the stabilization action of stabilizer muscle has been lost.^9,10^ After the familiarization phase, the participants performed random orders of Sahrmann five-level core stability test with abdominal muscle EMG testing and utilizing the PBU as their biofeedback. Participants were given a 2-min rest in between two test levels to reduce muscle post-activation potential carryover effects. Participants performed three trials for each level with at 1-min rest between trials. The average percentages of MVC derived from the three trials of each level were used in data analysis.

**Fig. 2.**
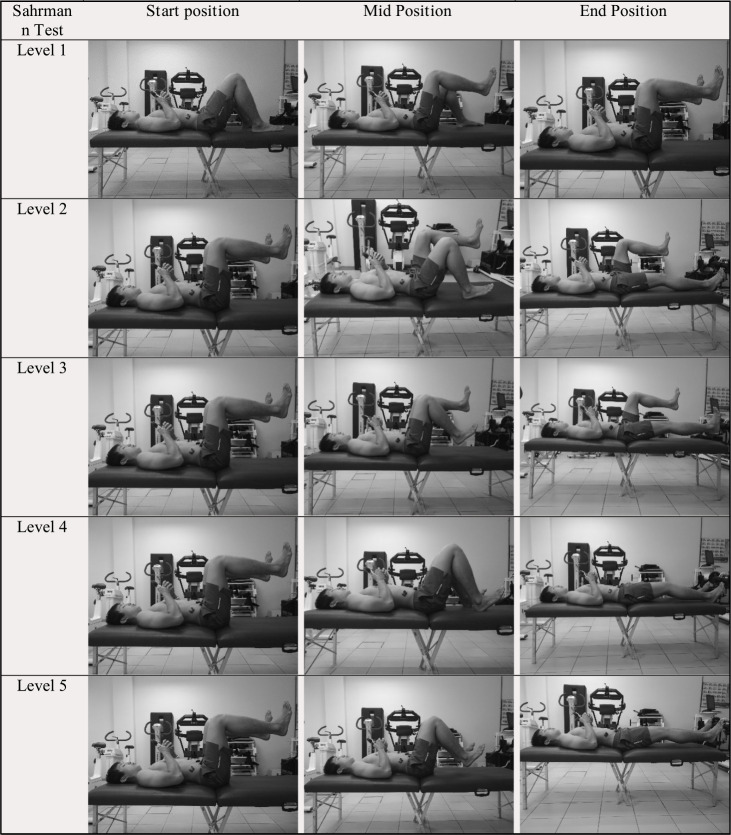
Illustration of the five levels of Sahrmann five-level core stability test.

The five levels of Sahrmann test are described in Table 1 and illustrated in Fig. 2.

**Table 1.** Five progressive levels of Sahrmann five-level core stability test.^9^

Level	Description
Level 1	The participant slowly raised one leg to a position of approximately 90^∘^ hip flexion with 90^∘^ knee flexion. The participant then attempted to bring the opposite leg also to the same position in the same manner.
Level 2	From the final position of the previous level, the participant slowly lowered one leg such that the heel contacted the ground/plinth. Then the leg slid out to fully extend the knee.
Level 3	From the end position of level 1, the participant slowly lowered one leg such that the heel reached approximate 12 cm above the ground. Then the leg slid out to fully extend the knee.
Level 4	From the final position of level 1, the participant slowly lowered both legs together such that the heels contacted the plinth. Then the legs slid out to fully extend the knees.
Level 5	From the final position of level 1, the participant slowly lowered both legs simultaneously such that the heels reached 12 cm above the ground. The legs then slid out to fully extend the knees.

### Data processing

The EMG signals generated during both MVCs testing and Sahrmann five-level core stability testing were analyzed and filtered using root-mean-square (RMS) technique (EMGworks 3.0, Delsys Analysis Software, Boston, USA). The MVC for each muscle was determined by calculating the peak EMG signal throughout the 5-s period of MVC. The starting and end of each EMG signal of the Sahrmann test were manually determined by assessing the muscle activities at baseline (rest periods), during the test, and at the end when the muscle activity returned to baseline.^20^ Lastly, the EMG activity during Sahrmann five-level core stability test was normalized by the MVCs for each muscle.

### Statistical analysis

Statistical analysis was carried out using IBM SPSS Statistics for Windows, version 25.0 (IBM, Armonk, NY). The normality of data was tested using the Shapiro–Wilk test. Categorical and continuous data were then analyzed using the most appropriate statistical tests based on data distribution. The Friedman two-way analysis of variance (ANOVA) by ranks was performed to test for the differences in EMG activities. Where a significant difference emerged, a multiple comparison procedure with the Wilcoxon signed-ranked test was used to test which pairwise differences were significant. A Bonferroni-adjusted alpha level was used to safeguard for the Type-1 error to be accepted as significant. The significance value for all was set at p<0.05.

### Sample size calculation

An *a priori* analysis was conducted for sample size calculation of repeated-measures ANOVA (within factors) using G∗Power for Windows, version 3.1.7.^21^ The effect size from past-related used was from  Ref. 22. The power analysis showed that F-test with an effect size of 0.328, alpha and statistical power equal to 0.05 and 0.95, respectively, with one group and five repeated measures yielded a sample size of 19 participants with an actual power of 0.96. A 15% sample size calculation adjustment for non-parametric Friedman test was added for the final sample size of this study to become 22 participants.^23^

## Results

### Comparison of EMG activity within five levels of Sahrmann five-level core stability test

Table 2 summarizes the data for the electromyographic activities of three abdominal muscles (MVC %) during the Sahrmann five-level core stability test. Statistically significant differences in the normalized EMGs of RA [$\chi^{2}$(4) = 64.80, p<0.001], EO [$\chi^{2}$(4) = 58.11, p<0.001], and TrA/IO [$\chi^{2}$(4) = 56.00, p<0.001] were observed between the five levels of Sahrmann five-level core stability test.

**Fig. 3.**
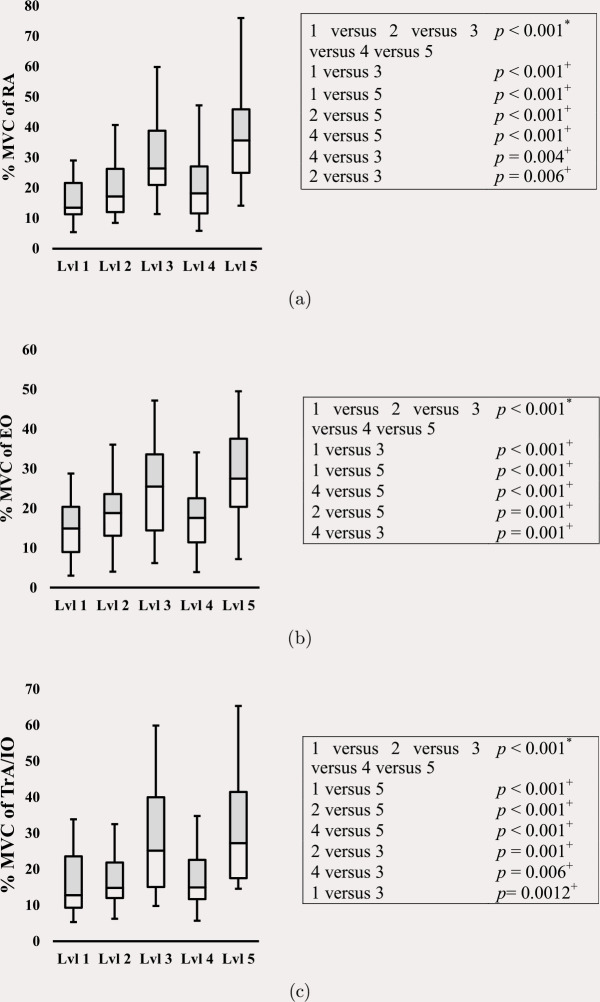
(a) Boxplot for MVC % of RA muscle between level (Lvl) 1 and level 5 of Sahrmann five-level core stability test; ^∗^Friedman test and ${}^{+}$Wilcoxon signed-rank tests with Bonferroni correction. (b) Boxplot for MVC % of EO muscle between Lvl 1 and Lvl 5 of Sahrmann five-level core stability test; ^∗^Friedman test and ${}^{+}$Wilcoxon signed-rank tests with Bonferroni correction. (c) Boxplot for MVC % of TrA/IO muscle between Lvl 1 and Lvl 5 of Sahrmann five-level core stability test; ^∗^Friedman test and ${}^{+}$Wilcoxon signed-rank tests with Bonferroni correction.

*Post-hoc* analysis using the Wilcoxon signed-rank tests with Bonferroni correction revealed that Sahrmann level-5 test elicits significantly greater EMG signals from all three abdominal muscles as compared to levels 1, 2, and 4 (p<0.001). No significant difference in all three abdominal muscle EMG signals was noted between Sahrmann levels 3 and 5. Sahrmann level-3 test yields significantly higher RA and TrA/IO muscle EMG signals compared to Sahrmann levels 1, 2, and 4. Sahrmann level-3 test demonstrated significantly higher EO muscle EMG signal than Sahrmann levels 1 and 4. No difference was noted in the EO muscle EMG signals between Sahrmann levels 3 and 2. The graphical representations of RA, EO, and TrA/IO muscle EMG signals are shown in Figs. 3(a)–3(c).

**Table 2.** Descriptive statistics of electromyographic activities of three abdominal muscles (MVC %) during Sahrmann five-level core stability test (N=22).

		Abdominal muscle activity (MVC %)		
Sahrmann	Muscles	Q1	Median	Q3
Level 1	RA	11.30	13.49	21.63
	EO	8.95	14.87	20.35
	TrA/IO	9.28	12.74	23.58
Level 2	RA	12.01	17.20	26.25
	EO	13.04	18.78	23.56
	TrA/IO	11.97	14.81	21.85
Level 3	RA	20.97	26.40	38.84
	EO	14.37	25.47	33.55
	TrA/IO	15.05	25.15	39.95
Level 4	RA	11.62	18.19	27.08
	EO	11.37	17.56	22.53
	TrA/IO	11.68	14.93	22.57
Level 5	RA	24.96	35.69	45.90
	EO	20.33	27.46	37.54
	TrA/IO	17.50	27.24	41.41

*Note*: RA: Rectus abdominis; EO: external oblique; TrA/IO: transversus abdominis/internal abdominal oblique; and MVC: maximum voluntary contraction.

### Comparison of EMG activity within muscles

This study also compared EMG activities within abdominal muscles (RA, EO, and TrA/IO) during each level of Sahrmann five-level core stability tests. No significant difference was found between RA, EO, and TrA/IO EMG activities during each level of Sahrmann five-level core stability test (p>0.05).

## Discussion

The purpose of this study was to evaluate the abdominal muscle activity (RA, EO, and TrA/IO) during different levels/maneuvers of Sahrmann five-level core stability test. The Sahrmann five-level core stability test was assumed to evaluate the abdominal muscle function as they act by isometrically contracting into flexion to maintain lumbar spine flexion by posteriorly tilting the pelvis against an assumed increasing resistance. The abdominal muscle activity levels are supposed to increase from Sahrmann levels 1 to 5.

The results of this study suggest that the abdominal muscle activity varies between the five different maneuvers of Sahrmann core stability test, with the highest abdominal muscle activities recorded during level 5, followed by level 3, level 4, level 2, and level 1. To our best knowledge, this is one of the few studies which explore abdominal muscle activity during non-heel contact and heel contact during leg lowering maneuvers. This study showed that the abdominal muscle activity was higher when heels do not contact the ground as noted in levels 3 and 5. A similar study by Gilleard and Brown^24^ showed that thigh unsupported leg lowering yields greater abdominal muscle activity than thigh supported with hands leg lowering. This shows less recruitment of abdominal muscles may be needed during supported lowering (levels 2 and 4) as compared to unsupported lowering (levels 3 and 5) because participants may have utilized the lower limb muscles to support the abdominal muscles to maintain the neutral lumbar curvature.

Our study also reveals similar abdominal muscle synergy pattern in unilateral leg lowering (level 3) and bilateral leg lowering (level 5). Our findings in this study are supported by those of Richardson *et al.*^25^ who also reported no significant difference in abdominal muscle activity between pelvic tilt with unilateral leg lowering and pelvic tilt with bilateral leg lowering. However, this study contradicts with the findings from Ref. 24 which observed lower RA activity but higher IO and EO muscle activities in bilateral leg lowering as compared to unilateral leg lowering. Shields and Heiss^26^ proposed two muscle synergy patterns during bilateral leg lowering. The first pattern included high RA, high EO, and low IO muscle activations, while the second pattern includes low RA, high EO, and low IO muscle activations. The Sahrmann level-5 bilateral leg lowering test differs from the conventional leg lowering test as the leg lowering maneuvers are started from 90^∘^ hip and knee flexion compared to conventional leg lowering test which started in a 90^∘^ hip flexion with knees extended, therefore different muscle synergy patterns may occur. During conventional leg lowering, when legs are lowered from a vertical position to a horizontal position, high external torque is created by the mass of the limbs (about 30% of the body weight) resulting in a great challenge to the abdominal muscles.^22,27^

This study has several limitations that needed to be addressed. First, the cross-talks between surface electrodes are minimized in this study by using standardized surface EMG electrode position as described by previous researchers.^14,15,16,17,18^ McGill *et al.*^15^ proposed the location of surface TrA/IO electrodes which accurately reflects the muscle activity of deep abdominals. However, the TrA/IO electrodes are still susceptible to cross-talk as they lie underneath superficial muscles.^20^ Furthermore, the recorded EMG signal from the TrA/IO electrode would come largely from IO rather than TrA because IO is more superficial than the TrA.^28^ Second, all participants in this study were healthy young men. The abdominal muscle activity pattern may differ between gender and among individuals with musculoskeletal conditions. Previous studies have reported significantly higher RA and EO muscle activities among women compared to their male counterparts.^29^ Additionally, higher RA and EO with lower IO muscle activities were reported among patients with low back pain compared to healthy individuals.^30^ Further exploration of abdominal muscle activity pattern during the Sahrmann core stability test among women and individuals with musculoskeletal conditions is warranted. Recommendations for future research include: (1) examining the abdominal muscle activity pattern of Sahrmann five-level core stability test in different populations, i.e., females and individuals with musculoskeletal conditions; and (2) investigating the effects of adding upper limb movements during performing Sahrmann five-level core stability test.

## Conclusion

To our best knowledge, this is one of the few studies which investigated the Sahrmann five-level core stability test on abdominal muscle activity. The results of this study showed that the abdominal muscle responds differently in supported (levels 2 and 4) or unsupported (levels 3 and 5) positions. Therefore, consideration should be taken in the future for the classification exchange in levels 3 and 4 of the Sahrmann five-level core stability test. Correspondingly, classification of the Sahrmann five-level core stability test into two smaller subcategories can also be considered, i.e., Poor (levels 1, 2, and 4) and Good (levels 3 and 5). As all the abdominal muscle sites were recruited below 40% of maximal isometric contraction, endurance benefit could be obtained with an appropriate number of repetitions. This may indicate that the Sahrmann five-level core stability test can be used as a screening tool and an abdominal endurance training regime.

## Acknowledgments

The authors would like to thank the Physiology Laboratory, Centre for Sports and Exercise Sciences, University of Malaya for providing data collection space and equipment. Thank you also to all subjects who participated in this study.

## Conflict of Interest

The author(s) have no conflicts of interest relevant to this paper.

## Funding/Support

This research did not receive any grants from any funding agencies.

## Author Contributions

The concept and designing of paper was carried out by all authors. Ebby Waqqash Mohamad Chan prepared the manuscript along with data collection and analysis. Eliza Hafiz and Mohamad Shariff A. Hamid helped in research design planning, results validation and interpretation. Ali Md. Nadzalan helped in data collection and research team training.
